# Increasing Bending Strength of Polycarbonate Reinforced by Carbon Fiber Irradiated by Electron Beam

**DOI:** 10.3390/polym15224350

**Published:** 2023-11-08

**Authors:** Yoshitake Nishi, Naruya Tsuyuki, Helmut Takahiro Uchida, Michael C. Faudree, Kouhei Sagawa, Masae Kanda, Yoshihito Matsumura, Michelle Salvia, Hideki Kimura

**Affiliations:** 1 Graduate School of Engineering, Tokai University, Hiratsuka 259-1292, Japan; west@tsc.u-tokai.ac.jp (Y.N.); 6bajm036@mail.u-tokai.ac.jp (N.T.); helmutuchida@tokai.ac.jp (H.T.U.); sagawa.kouhei@tokai.ac.jp (K.S.); kanda@isc.chubu.ac.jp (M.K.); ncc1701d@keyaki.cc.u-tokai.ac.jp (Y.M.); kimura@tokai-u.jp (H.K.); 2 Graduate School of Science & Technology, Tokai University, Hiratsuka 259-1292, Japan; 3 Laboratoire de Génie Electrique et Ferroéléctricité (LGEF), INSA Lyon, CEDEX, 69621 Villeurbanne, France; 4 Ecole Centrale de Lyon, CEDEX, 69134 Ecully, France; michelle.salvia@ec-lyon.fr; 5 Kanagawa Institute of Industrial Science and Technology (KISTEC), Ebina 243-0435, Japan; 6 Faculty of Liberal Arts and Science, Tokyo City University, Yokohama 224-8551, Japan; 7 Center of Applied Superconductivity & Sustainable Energy Research, Chubu University, Kasugai 487-8501, Japan

**Keywords:** thermoplastic, polycarbonate, carbon fiber, electron beam, dangling bonds, bending strength

## Abstract

In an interlayered carbon fiber-reinforced polycarbonate polymer (CFRPC) composite composed of three sized of CF plies, alternating between four PC sheets, designated [PC]_4_[CF]_3_, and a new process of activating CF cross-weave cloth plies directly on both sides with homogeneous low-energy electron beam irradiation (HLEBI) before lamination assembly and hot pressing at 6.0 MPa and 537 K for 8 min was produced. Experimental results show that a dose of 215 kGy of HLEBI raised the bending strength, σ_b_, at each experimental accumulative probability, *P*_a_, with the *σ*_b_ at a median *P*_a_ of 0.50, increasing by 25% over that of the untreated sample. Three-parameter Weibull analysis showed that when quality can be controlled, a dose of 215 kGy of HLEBI can raise the statistically lowest bending strength, σ_s_, at *P*_a_ = 0 (94.3 Mpa), with a high correlation coefficient. This is because, although it had a higher bending strength than that in the other experimental conditions, the weakest sample of the 215 kGy data set had a much lower σ_b_ value than that of the others. Electron spin resonance (ESR) of the CF showed that naturally occurring dangling bonds in CF were increased at 215 kGy. Charge transfer to the PC occurs, apparently generating stronger bonds, which are possibly covalent, resulting in enhanced adhesion at the CF–PC interface.

## 1. Introduction

It is imperative to transition to a highly sustainable society by increasing the utilization of recyclable materials to live in increasing harmony with nature. Using the conventionally manufactured carbon fiber-reinforced polymer (CFRP) consisting of sized CFs and a high-strength thermoset (TS) epoxy resin matrix has been the accepted practice and has been applied in aircraft fuselages, spacecraft, wind turbine blades, and sports equipment, to name a few applications. Epoxy has a higher strength and better interfacial adhesion to CF than thermoplastics (TPs). If CFRP is not used, automobile parts or concrete columns can corrode, leading to insufficient bending strength [1]. Nevertheless, significant problems with epoxies are known to include their non-recyclability, due to their cross-linked molecular structure and their long curing time, which requires higher energy consumption. Moreover, epoxies have poor toughness, and higher water absorption than that of TPs, which results in hydrolysis and plasticization in long-term service environments. TPs, on the other hand, have increased resistance to cracking, cheaper material costs, and shorter production times. In addition, TPs are a promising alternative to epoxies due to their recyclability, allowing them to be melted and reformed repeatedly, reducing scrap and contributing to a cleaner and more sustainable environment.

**Figure 1:**
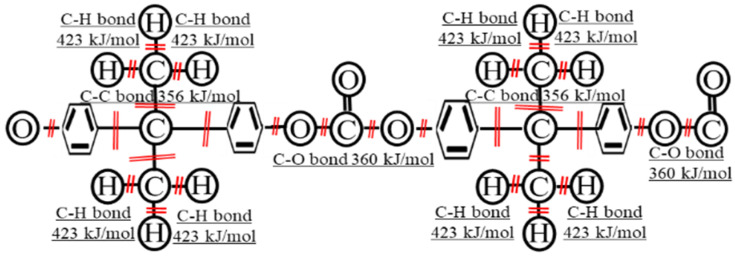
Constitutional formula of PC with dangling bond sites and their approximate. Dissociation energies [4,39] brought about by charge transfer from HLEBI-activated CF.

It follows that polycarbonate (PC) constructed from hydrogen and carbon, as shown in Figure 1, is a commonly used thermoplastic (TP) that is a highly transparent engineering plastic with more than 150 times the mechanical strength of tempered glass. PCs are commonly used for protective items, including goggles and face shields, helmets, protective glazing for buildings, windows, household appliances, and covers for electronic equipment, because they are lightweight, have superior properties such as processability and impact resistance [2,3], and can withstand severe weather conditions. PC has a maximum continuous service temperature of 423 K (140 °C) [4]. PC is commonly used for aircraft windows, and also has a short solidification duration that is less than 1/10th of that of epoxy, thus reducing the required energy requirements for fabrication. PC has a higher tensile strength than that of several other polymers, at 55 to 65 Mpa [4], and an excellent Izod impact strength at 19 J [5]. The drawbacks of PC are that it scratches easily, that UV-grade PC is required for outdoor use, and that PC should not be used in contact with alcohols or strong alkalis.

CF itself has excellent mechanical properties including a reported 4.4 GPa tensile strength and a 377 GPa tensile modulus [6], decent resistance to fatigue, durability, and a greater corrosion resistance than that of synthetic fibers [7]. Moreover, CF has an extremely small diameter of ~6 μm, allowing broad and intricate surface area contact with polymers. However, it is challenging for CF to adhere to TP owing to its non-polarity, poor wettability, and hydrophobicity [8], of both CF and TP. In addition, poor adhesion can be caused by the smoothness of the CF surface. Hence, numerous studies have focused on treating CF to increase adhesion at the interface [9,10,11,12,13,14,15,16,17,18,19,20,21,22,23,24,25,26,27]. Acidic modification has been utilized to increase polar groups [9,10] and interfacial friction at the CF surface via enhancing interlocking between the fiber and matrix [11], but the disadvantages of this approach are decreased strength [12] from surface damage, and the weight loss of the CF [13]. For the adhesion of recycled CF to PP resin, the application of 932 K (650 °C) of superheated steam for 1 hr has been performed to attach oxygen functional groups to CF [14]. The plasma surface modification of CF has been extensively studied [15,16,17,18,19] and has worked to increase the interlaminar shear strength of CFRP. Several other studies have involved the introduction of polar groups to the CF surface [20,21,22,23], including -NH_2_, -OH, and -COOH, along with the strengthening of CF itself [20]. Rare-earth particle attachment to CF has also found success in enhancing composite mechanical properties [22,23]. High-energy irradiation techniques [24,25], by activating CF crystal lattice sites and enhancing surface roughness, such as in Ar+ [26] and Co^60^ γ-ray [27] irradiation, have increased its mechanical properties.

It follows that low-voltage electron beam irradiation (HLEBI) has been used to improve the mechanical properties of numerous materials [2,28,29,30,31,32,33,34,35,36,37,38]. For the PC macromolecule, HLEBI generates dangling bonds at bonding sites with the lowest dissociation energies, as shown in Figure 1. Dangling bonds between C-C and C-O are expected to most easily form with their dissociation energies of 356 kJmol^−1^ and 360 kJmol^−1^, respectively [4,39]. Since the CF is activated with HLEBI, charge transfer is expected to occur in the PC at the interface generating strong bonds with CF, preventing CF pull-out and ply delamination. Studies on electron beam treatment for recyclable TP polymers without CF are few in number [2,28,29,30] but include those on PC [2], polyurethane (PU) [28,29], and polypropylene (PP) blends [30]. For CFRTP PP, tensile strength and Young’s modulus were increased with an EB dose from 100 to 400 kGy [31] while, for TP PEEK (polyetheretherketone) CFRP specimens, impact strength was increased by 56% at a low accumulative probability, indicating increased reliability and safety by the possibility of strengthening the weakest samples in the data [32]. As for CF itself, electron beam treatment was found to strengthen CF embedded in the polymer matrix [33], and produce an excellent tensile strength and electrical conductivity of CF when the electron beam was applied prior to heat treatment during fabrication [34]. Also, the fracture stress of CF in single-fiber testing was raised to over 10 GPa at a high accumulative probability using a 112 Mrad electron beam, which also raised the Weibull modulus and aircraft design stress [35]. Enhancements were attributed to the migration of unstable terminated C atoms to vacant sites that would act as crack origins, dulling sharp crack tips, and the relaxing of the stress concentration [35]. However, higher electron beam doses were found to reduce the mechanical properties of CF: this is if interstitial atoms are formed between the hexagonal graphitic planes or if excess dangling bonds are generated within graphitic planes [35].

As far as the authors know, there have been few or no studies on applying HLEBI to strengthen interlayered CFRTP structures. Therefore, our research has focused on strengthening alternating CF-TP interlayered samples by applying HLEBI prior to assembly and heating by hot press to either the TP plies [36] or CF plies [37], or after molding to finished samples [38] to raise the mechanical properties. For an interlayered composite of four plies of TP polyphenylene sulfide and three plies of CF with layup PPS-CF-PPS-CF-PPS-CF-PPS labeled here as [PPS]_4_[CF]_3_, HLEBI of 5 kGy applied to both sides of PPS plies prior to assembly and hot press was found to raise impact values, particularly those of the lowest strength at low *P*_f_, showing that an increase in safety and reliability is possible [36]. Direct HLEBI activation of 0.22 Mgy to CF plies before being put together with PP plies (the same layup as above, but with PP) slightly increased the bending strength of a [PP]_4_[CF]_3_ interlayered composite, by approximately 6% [37]. For an interlayered composite composed of nine CF plies alternating between 10 TP polyamide plies designated [TPA]_10_[CF]_9_, when applying HLEBI, increasing acceleration voltage from the usual 170 kV [32,36,37] to 250 kV for the finished samples, the impact strength could be increased by 25 to 27% compared to the untreated sample [38].

On the other hand, strong adhesion between CF, and the widely used PC TP, with its strong impact resistance, versatility, transparency, toughness and recyclability, would be highly sought after. Up to now, investigating interlayered [PC]_4_[CF]_3_ CFRTP composite and increasing its strength by any method including HLEBI has not been investigated. Therefore, this study focuses on the effect of HLEBI directly to CF prior to lamination assembly and hot press on the bending properties of an interlayered PC/CF composite. Of course, investigating other properties such as tensile, lap shear, open hole compression, edge delamination strength (EDS), impact, and compression after impact (CAI) properties are required to approve materials for aerospace and other applications, but were beyond the scope of this study. One thing to note is that the bending test depends on flaws in the outer plies in tension and compression, while the tensile test depends on flaws throughout sample thickness; hence, bending strengths are typically higher than tensile strengths. These tests should be considered for future studies. The goal of this study is to increase the mechanical property of bending strength by treating CFs directly with HLEBI for [PC]_4_[CF]_3_ samples. This is carried out with the ultimate aim of fabricating layered CF/PC composites as a viable option for the application of recyclable PC in automobiles, aerospace, sports equipment, and building construction, to name a few examples.

## 2. Experimental Procedure

### 2.1. Materials and Fabrication of [PC]_4_[CF]_3_ Samples

**Figure 2:**
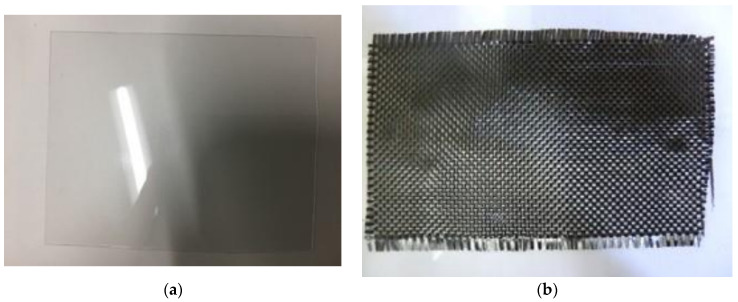
Photos of transparent PC sheet (**a**), and CF cloth (**b**). For PC sheet, the fluorescent ceiling light in the lab reflects off the PC, showing the shiny surface.

**Figure 3:**
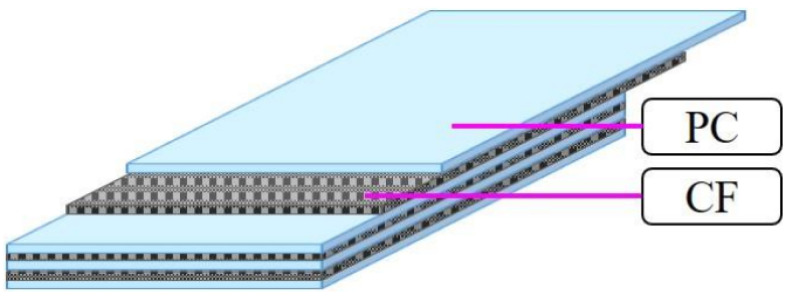
Illustration of [PC]_4_[CF]_3_ sample (not drawn to scale).

Samples were constructed of a PC sheet and CF cloth, as shown in Figure 2a,b and Figure 3. As illustrated in Figure 3, three sizes of CF plies were assembled between 4 PC sheets with a ply order of [PC-CF-PC-CF-PC-CF-PC], the layup designated here as “[PC]_4_[CF]_3_”. The 7-layer layup was chosen to make finished samples after molding that are 2 mm in thickness, in accordance with Japanese Industrial Standard JIS K 7074 [40]. Next, the solidification of the layered structure was performed by one-directional hot press (IMC-185A, Imoto Machinery Co., Ltd., Tokyo, Japan) under 6.0 Mpa at 537 K (264 °C) for 8 min. Hot-press molding parameters were adjusted for maximum cohesion between plies and to achieve the cleanest samples. The CF used was plain cross-weave (TR3110M: Mitsubishi Rayon Ltd., Tokyo, Japan) plies with a 230 µm thickness (before molding), and areal weight listed as 198 to 200 gm^−2^ [41]. The presence of CF epoxy sizing and its composition was confirmed by proton-NMR (AVANCE500, Neutron Magnetic Resonance, Shimazu, Kyoto) [37]. The polymer used was a TP PC sheet (Sugawara Kougei Ltd., Tokyo, Japan) with a 0.3 mm thickness. Each PC sheet (110 mm × 170 mm × ~286 µm) was hot pressed by PC particles (3 g) under 15 Mpa at 418 K for 3 min.

The dimensions of the finished samples (thickness, width, length) were as follows: 2.0 mm × 10 mm × 80 mm. The CF volume fraction, *V*_f,CF_, of molded [PC]_4_[CF]_3_ samples was about 0.55. The ply thickness of finished samples is presumed to be 2.0 mm/7 = 286 µm.

### 2.2. Condition of HLEBI

A portion of the [PC]_4_[CF]_3_ samples had CF plies treated with homogeneous low-energy electron beam irradiation (HLEBI) curtain processor (Type CB175/15/180L, Energy Science, Inc., Woburn, MA, Iwasaki Electric Group Co., Ltd., Tokyo, Japan) on both sides before assembly with untreated PC sheets, and subsequent hot press. The total HLEBI doses investigated were as follows: 0 (untreated), 43, 129, 215, 301 and 430 kGy. The experimental HLEBI dose range (0 to 430 kGy) was chosen by conducting preliminary bending tests to determine if a maximum bending strength can be achieved above untreated and to what degree, and at what higher doses, would bending strength would drop off from excess HLEBI. During treatment, temperature was controlled so not to exceed 323 K. Given the reported CF density of 1760 kgm^−3^, the penetration depth, *D*_th_, was 123 μm into both sides of each 230 μm thick CF ply, penetrating throughout the ply’s thickness [42]. A detailed description and parameters of the HLEBI treatments employed can be found in [43].

### 2.3. Bending Tests

Finished samples were subjected to two types of 3-point bending tests. The first was non-destructive bending test standard JIS K 7074 [40], which was carried out on the [PC]_4_[CF]_3_ bending specimens [*l* × *w* × *t*] = 80 × 10 × 2.0 mm at very low deformations, measuring displacement using a red laser to obtain initial elastic bending moduli (μ_i_). The warm-up time of the laser setup was ~1 h. The span and midpoint were 40 mm and 20 mm. Weights of 100, 200 and 400 g were suspended from the center of the sample with a homemade hook device. A voltmeter recorded the displacement. When taken through the zero point, the elastic modulus can be obtained; therefore, both sides of the specimen were tested: the “tension” and “compression” sides are arbitrary. Carefulness was needed to prevent any external vibrations.

Secondly, to obtain ultimate bending strength (*σ*_b_), the same samples were put under 3-point bending with a tester (IMADA Co., Ltd., DPU-50N/MX-500N/GA-10N) according to Japanese Standard JIS K 7074 [40] at ambient temperature. The span and midpoint were 40 mm and 20 mm, respectively, while the head speed was 5 mm min^−1^. Bending stress–strain (*σ* − *ε*) curves were recorded based on crosshead displacement while being simultaneously filmed with a video recorder for confirmation. The *σ*_b_ and μ_i_ were calculated according to JIS K 7074 [40].

### 2.4. Accumulative Probability

Bending strengths, *σ*_b_, of samples are ranked according to their accumulative probability, *P*_a_, where 0 < *P*_a_ < 1.0, the higher *P*_a_ being the strongest [44]. This is a statistical analysis calculation commonly used to assess the reliability and safety of manufactured parts using Equation (1) [44]:

*P*_a_ = (*i* − 0.3)/(*N*_s_ + 0.4) (1)

The *i* is the rank order integer where the higher number is the stronger, while *N*_s_ is number of samples in a data set. *P*_a_ and *P*_e_ will represent accumulative probability for bending strength, and initial elastic bending modulus, respectively. For simplicity, “initial elastic bending modulus” will be referred to as “elastic modulus”.

## 3. Results

### 3.1. Effects of HLEBI to CF on Elastic Modulus of [PC]_4_[CF]_3_ Samples

**Figure 4:**
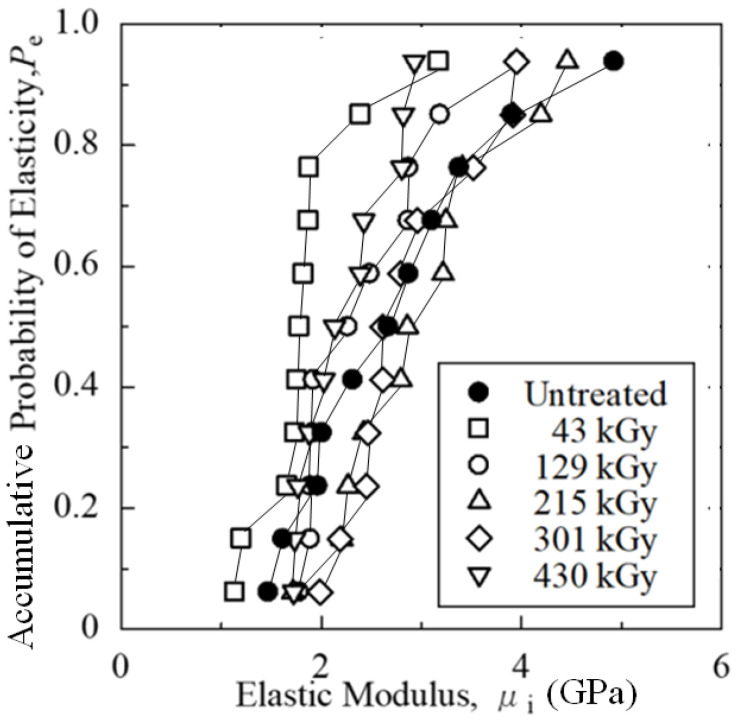
Accumulative probability, *P*_e_ vs. initial elastic bending modulus, μ_i_ (GPa) for untreated and HLEBI-treated [PC]_4_[CF]_3_ samples.

Figure 4 shows experimental results of accumulative probability, *P*_e_, vs. elastic modulus, μ_i_, at extremely low strains for untreated and HLEBI-treated [PC]_4_[CF]_3_ samples of 43, 129, 215, 301 and 430 kGy doses, respectively. The optimum appears to be the 215 kGy dose exhibiting 6 maxima out of the 11 sample data set at 4.20, 3.25, 3.22, 2.85, 2.80, and 2.20 GPa at *P*_e_ = 0.85, 0.68, 0.59, 0.50, 0.41, and 0.15, respectively. The second strongest was the 301 kGy data set having five maxima, since the elastic bending modulus at *P*_e_ = 0.15 was equal to that of the 215 kGy data set at 2.20 GPa. At high *P*_e_ = 0.94, the untreated sample had the highest overall μ_i_ at 4.92 GPa, although the 215 kGy samples had higher μ_i_ at most *P*_e_.

**Figure 5:**
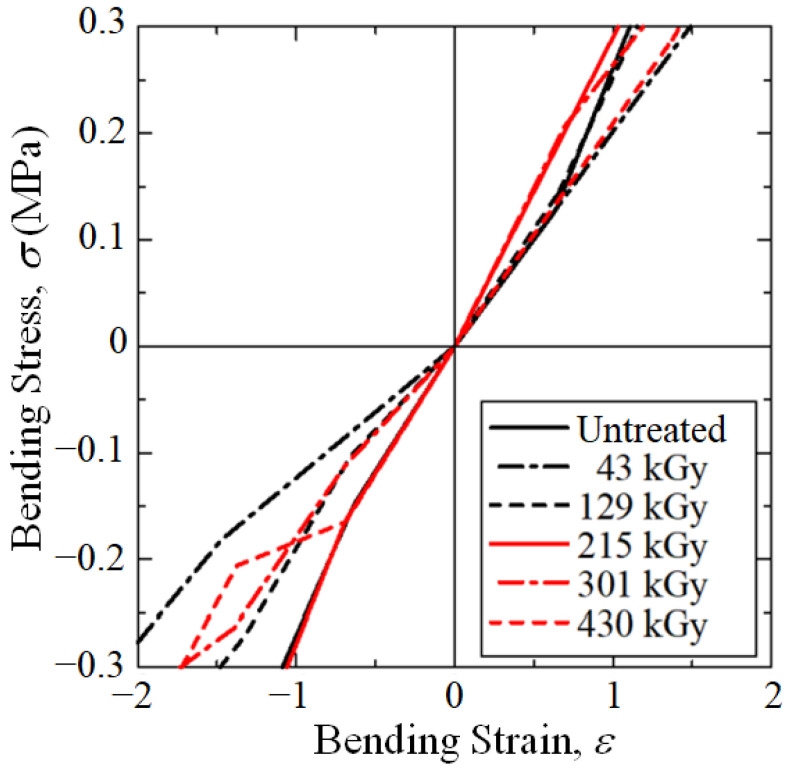
Bending stress–strain curves at median *P*_e_ = 0.50.

Figure 4 shows that, at median *P*_e_ = 0.50, the 215 kGy dose resulted in the highest μ_i_ at 2.85 GPa. In addition, Figure 5 shows for the bending stress–strain curves that, at median *P*_e_ = 0.50, the 215 kGy HLEBI dose (solid red line) exhibited the highest elastic modulus for tension and compression through the zero point.

Note, the low dose of 43 kGy HLEBI resulted in a decrease in elastic modulus at all accumulative probabilities (Figure 4) and the lowest at median *P*_e_ = 0.50 (Figure 5).

### 3.2. Effects of HLEBI to CF on Bending Strength of [PC]_4_[CF]_3_ Samples

**Figure 6:**
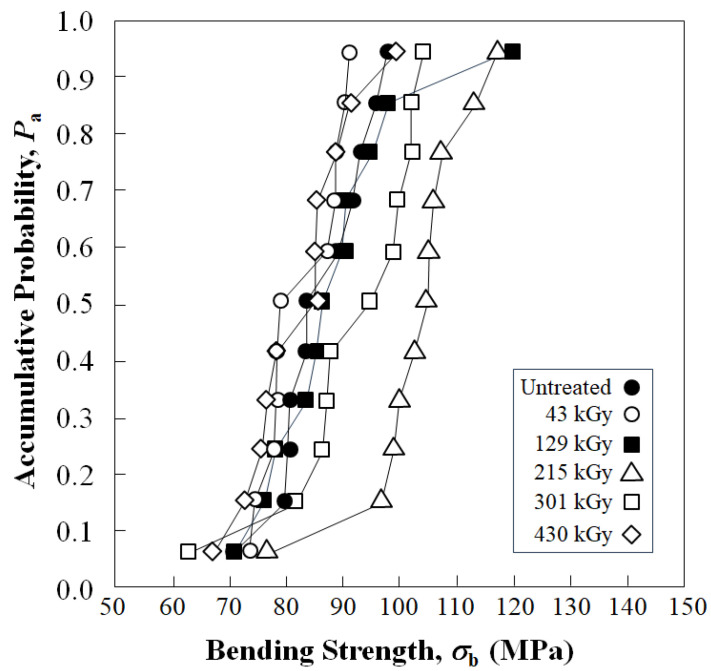
Accumulative probability, *P*_a_, vs. bending strength, *σ*_b_, for untreated and HLEBI-treated [PC]_4_[CF]_3_ samples.

Figure 6 shows experimental results of accumulative probability, P_a_, vs. bending strength, σ_b_, of untreated and HLEBI-treated [PC]_4_[CF]_3_ samples for 43, 129, 215, 301 and 430 kGy doses.

HLEBI doses of 215 or 301 kGy were found to raise bending strength values, σ_b_, at all *P*_a_ above 0.15. Although the weakest sample in the 215 kGy data set (*P*_a_ = 0.06) had a much lower σ_b_ (77 MPa) than the others (~95 to ~120 MPa), the σ_b_ at *P*_a_ = 0.06 was still higher than that of the untreated sample (71 MPa). Figure 6 shows that 215 kGy appeared to be at or near optimum, raising the strength by (77, 105, 117 MPa) 8.4%, 25%, and 19% over that of the untreated sample (71, 84, 98 MPa) at low, median, and high *P*_a_ = 0.06, 0.50, and 0.94.

Note that for one data point at high *P*_a_ above 0.94, the 129 kGy samples had a slightly higher σ_b_ (120 MPa) than that of 215 kGy, similar to the μ_i_ results in Figure 4. However, the 215 kGy samples had a much higher σ_b_ than 129 kGy, overall, at all *P*_a_ below 0.90.

In addition, Figure 6 shows that 301 kGy HLEBI raised σ_b_ at all *P*_a_ above 0.15, but to a lower degree than 215 kGy. Also, the lower HLEBI dose of 43 kGy slightly dropped σ_b_, whereas it was found to increase its strain at most fracture probabilities, *P*_a_ (not shown). On the contrary, the 129 kGy dose slightly raised σ_b_ at a *P*_a_ above 0.30, whereas it slightly dropped its strain and fracture energy at *P*_a_ less than 0.90.

**Figure 7:**
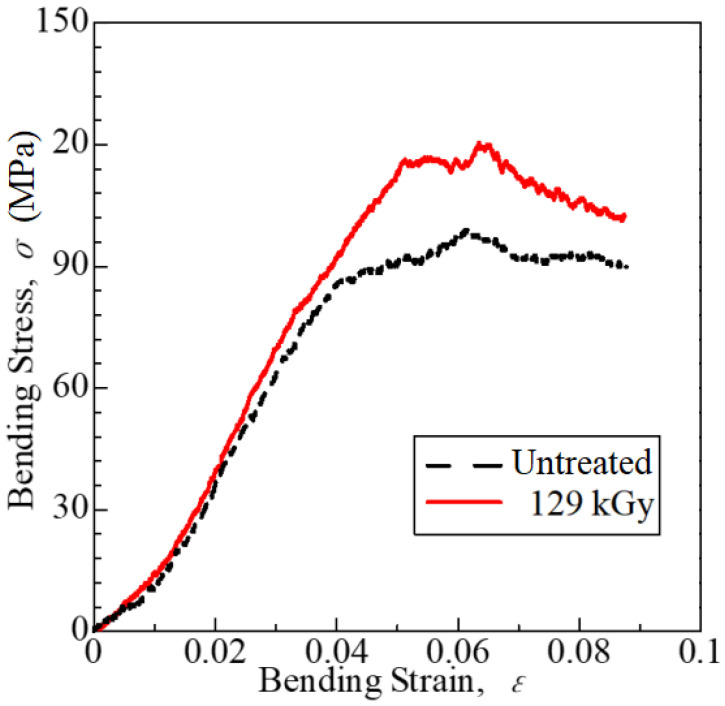
Representative bending stress–strain curves: untreated (black dotted line) and 129 kGy samples (red line) at *P*_a_ = 0.94 are shown.

Representative bending stress–strain curves for the [PC]_4_[CF]_3_ samples are shown in Figure 7. It compares untreated and 129 kGy conditions at *P*_a_ = 0.94. The HLEBI apparently increases adhesion between the PC and CF plies for a higher yield strength, increasing the maximum bending strength.

**Figure 8:**
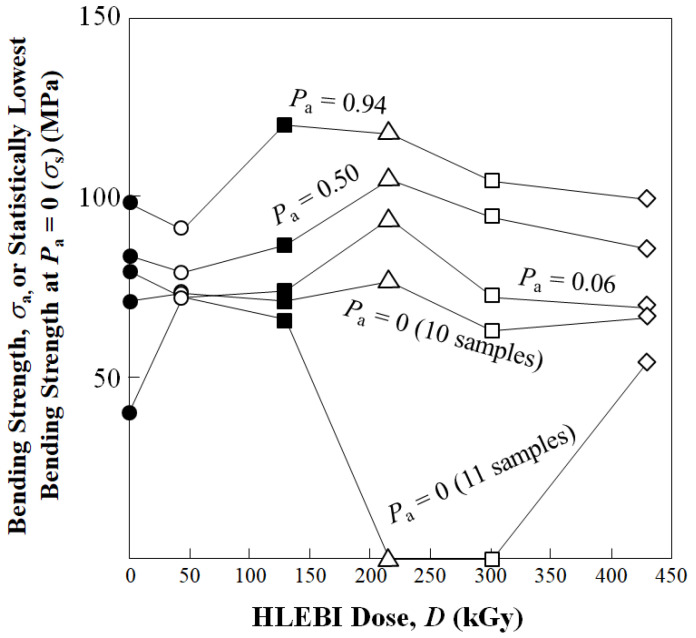
Changes in bending strength (MPa) at low, median, and high accumulative probabilities. (*P*_a_) of 0.06, 0.50 and 0.94, respectively, together with statistically lowest *σ*_s_ (*σ*_b_ at *P*_a_ = 0) for 10-sample and 11-sample data sets.

Figure 8 shows changes in σ_b_ at low, median and high *P*_a_ of 0.06, 0.50, and 0.94 for each experimental condition in Figure 6, again showing that the optimum appears to be at or near 215 kGy. The lower two plots are three-parameter Weibull calculations described in the next section.

## 4. Discussion

### 4.1. Three-Parameter Weibull Calculation for Statistically Lowest Bending Strength, σ_s_, at P_a_ = 0

Calculation with the three-parameter Weibull equation was carried out to determine the statistically lowest bending strength *σ*_s_ at *P*_a_ = 0 as a function of HLEBI dose. Three-parameter Weibull estimation is typically applied for quality control (QC). When it is assumed that the statistical equation is applicable to the experimental *σ*_b_ value, the *P*_a_ is dependent on the risk of rupture [45,46,47]. For predicting the required strength of new structural materials, the *σ*_s_, coefficient, *m* and constant (*σ*_III_) are the important parameters. The equation is

*P*_a_ = 1 − exp[−([*σ*_b_ − *σ*_s_]/*σ*_III_)*m*] (2)

In linear form, Equation (2) is

ln(−ln(1 − *P*_a_)) = *m*ln(*σ*_b_ − *σ*_s_) − *m*ln*σ*_III_ (3)

**Figure 9:**
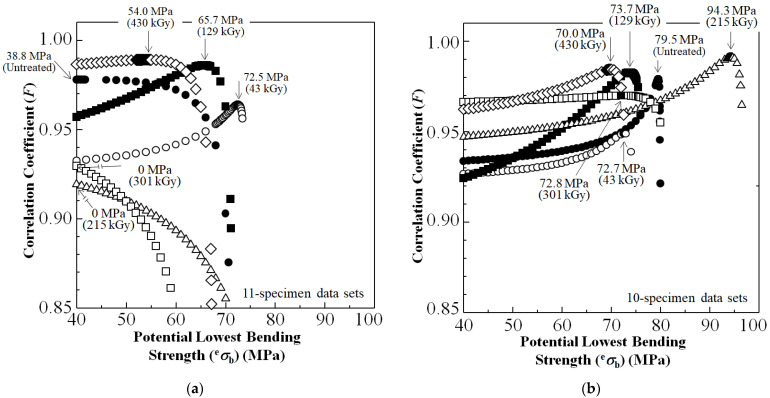
Changes in correlation coefficient (*F*) versus potential lowest *σ*_b_ value (^e^*σ*_b_) for untreated, and HLEBI-treated (to the CF) [PC]_4_[CF]_3_ samples for (**a**) 11-sample data sets, and (**b**) 10-sample data sets. The lowest bending strength, *σ*_s_ (arrows), is determined at maximum *F*.

To estimate the *σ*_s_ at *P*_a_ = 0, Equation (3) is iterated until the correlation coefficient, *F*, reaches a maximum. Figure 9 shows the resulting plots of *F* against potential *σ*_b_ value (^e^*σ*_b_), for untreated and HLEBI-treated [PC]_4_[CF]_3_ samples.

Figure 9a shows that, for the 11-sample data sets, applying the low 43 kGy dose resulted in the highest *σ*_s_ at *P*_a_ = 0 at 72.5 MPa, a 87% improvement over untreated at 38.8 MPa. Also, the 129 kGy HLEBI resulted in *σ*_s_ of 65.7 MPa, a 69% increase over untreated.

However, looking at Figure 6, the weakest samples in the data sets at *P*_a_ = 0.06 had significantly lower *σ*_b_ than the others; hence, the *σ*_s_ are calculated, eliminating the *P*_a_ = 0.06 samples. This is a common practice in industry that increases the reliability and safety of the parts; if the very lowest *P*_a_ samples have a much lower strength than the others, they can be omitted and remolded if the polymer is recyclable TP. Therefore, Figure 9b shows that, when quality can be controlled in this way, σ_s_ of all data sets are increased. Namely, the σ_s_ of the 10-specimen 215 kGy data set at 94.3 MPa is increased 18% over the untreated at 79.5 MPa to increase reliability and safety.

### 4.2. Effects of HLEBI on ESR Signals and σ_b_

**Figure 10:**
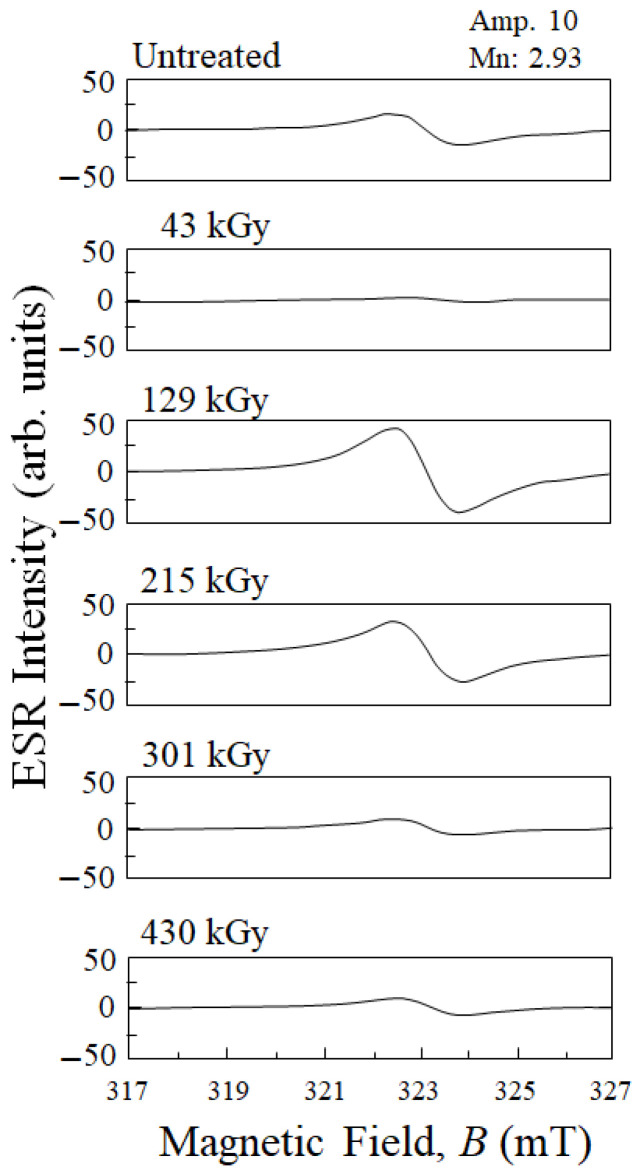
ESR signals of CF when untreated and treated with each HLEBI dose.

To assess action of dangling bonds in CF enhancing adhesion with PC, Figure 10 shows the ESR signals of untreated and HLEBI-treated CF. The untreated CF shows a peak whose inflection point resides at 323 mT, reported to appear due to naturally occurring dangling bonds in CF [32]. At 43 kGy, the dangling bond density of CF is generally reduced to zero as the peak disappears. This is probably due to the migration of unstable terminated C atoms to vacant sites as mentioned earlier [35], possibly increasing the inertness of the CF for lower adhesion to PC, reducing bending strength. However, interestingly, dangling bond density is sharply increased at 129 kGy and slightly reduced, but still high, at 215 kGy. This could be due to the generation of optimum density of vacant sites, i.e., dangling bonds for adhesion to PC in the form of strong bonds. Prior to this analysis, HLEBI was reported to decrease dangling bond density in CF; however, this was with the strong dose of 430 kGy [32], which agrees with the data from this study in Figure 10. This is a new finding: that at the intermediate doses of 129 kGy and 215 kGy, dangling bond density in CF can be increased over that of the untreated. Dangling bonds generated at 215 kGy apparently resulted in strong adhesion at the CF/PC interface to increase the σ_b_ values.

It follows that higher HLEBI doses of 301 or 430 kGy decreased CF dangling bond density to values lower than those of the untreated, resulting in lower *σ*_b_ values. This can be explained by excess charge transfer from the activated CF to the PC, weakening the PC structure with exchange interaction with trace gasses at the interface lowering ESR dangling bond signal.

It is possible that different types of dangling bonds are involved. As mentioned earlier, 43 kGy HLEBI annihilates the ESR peak. When the spontaneous dangling bonds are made to be metastable, it could explain their annihilation. It can be deduced that the 129 kGy and 215 kGy HLEBI generate a new or different type of dangling bond than the naturally occurring bonds, on surface and inside of CF, working to increase adhesion to the PC and preventing CF-PC pull-out and ply delamination. However, investigating the types of dangling bonds is beyond the scope of this study.

Comparing Figure 6 and Figure 10, *σ*_b_ appears to be generally related to the dangling bond density (peak height) of the CF. From an HLEBI of zero to 43 kGy, dangling bond density is reduced and *σ*_b_ at *P*_a_ = 0.06, 0.50 and 0.94 are reduced. From 43 to 129 kGy, dangling bond density sharply increases and *σ*_b_ are increased. From 129 to 215 kGy, dangling bond density slightly decreases and *σ*_b_ are decreased (at *P*_a_ = 0.94) and increased (at *P*_a_ = 0.06 and 0.50). From 215, to 301 and 430 kGy, dangling bond density is reduced and *σ*_b_ are reduced. Slight variation could be due to factors such as bonding with PC at the PC/CF interface, type of dangling bond (mentioned earlier), or excess radiation damage at higher doses along with contaminations by the residual gas of PC irradiated and the atmosphere that prevent bonding at the CF/PC interface.

**Figure 11:**
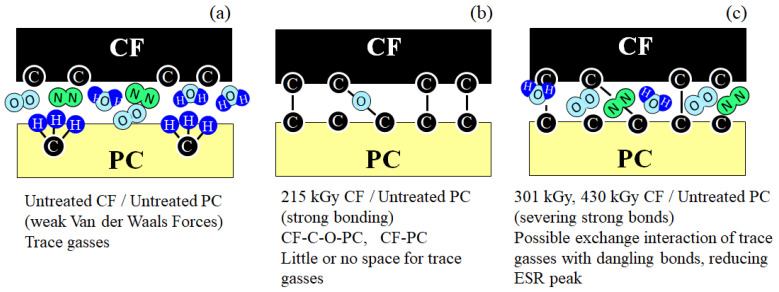
Schematic drawings of bonding states of CF/PC interface of [PC]_4_[CF]_3_ samples for (**a**) untreated, (**b**) 215 kGy, and (**c**) higher doses of 301 or 430 kGy HLEBI.

Figure 11 shows that the deduced mechanism of strength increases by 215 kGy HLEBI at the CF/PC interface. Figure 11a illustrates that, for untreated samples, trace atmospheric gas molecules of water, nitrogen, and oxygen (H_2_O, N_2_, O_2_) most likely exist at the CF/PC interface, producing weak Van der Waals attractive forces as CF-(H_2_O, N_2_, O_2_)-PC. A dominant mechanism is apparently mechanical friction between PC and CF with a lower CF pull-out and delamination resistance, bringing about a lower bending strength for untreated samples.

However, Figure 11b illustrates for 215 kGy HLEBI, that the generation of strong bonds CF:C:O:C:PC and CF:C:C:PC, which are possibly covalent, at the CF/PC interface, resulting in enhanced adhesion around the CF circumference, and prevents fiber pull-out and ply delamination. This apparently results in a significantly reduced quantity of gas molecules between the CF and PC, if the strong bonds block any space at the interface. In addition, as mentioned earlier, CF itself is reported to be strengthened by the HLEBI [32].

But, Figure 11c shows that, at higher HLEBI doses of 301 and 430 kGy, strong bonds can be severed at the interface, lowering strength due to radiation damage from the excess HLEBI.

The CF hexagonal structure itself will have increased entropy with excess dangling bonds, possibly undergoing exchange interaction with trace gasses, resulting in a hindered adhesion with PC. For these reasons, carefulness is highly recommended to adjust for optimal HLEBI dose when applying for practical purposes.

This study focused on the effect of HLEBI on the bending properties of [PC]_4_[CF]_3_ composites at room temperature. Effects of long-term service environments and loading on the bonding state of the CF/PC interface such as aging, fatigue, temperature, sun exposure, water, salt water environments, and fatigue were beyond the scope of this study. These should be investigated for practical situations for maximum safety and reliability. Nevertheless, this study shows that the bending strength of a [PC]_4_[CF]_3_ interlayered composite can be increased by an optimum HLEBI dose to the CF plies prior to lamination assembly and hot press.

## 5. Conclusions

It is always advantageous to obtain a strong bond between carbon fiber (CF) and the difficult-to-adhere thermoplastic polycarbonate (PC) since PC is recyclable and beneficial for a sustainable environment. Therefore, a new process for PC/CF composites of activating sized CF cross-weave plies directly with homogeneous low-energy electron beam irradiation (HLEBI) on both sides prior to lamination assembly and hot press under 6.0 MPa at 537 K for 8 min after HLEBI was found to increase the bending strength of composite samples composed of three CF plies between four PC sheets, [PC]_4_[CF]_3_.

1. Experimental results showed that the 215 kGy HLEBI dose appeared to be at or near optimum, raising bending strength at all accumulative probabilities, *P*_a_. At low, median, and high *P*_a_ of 0.06, 0.50, and 0.94, bending strength was increased by 8.4%, 25%, and 19% from 71, 84, 98 MPa for untreated, to 77, 105, 117 MPa for the 215 kGy samples.
2. Three-parameter Weibull analysis showed that, when quality can be controlled, 215 kGy HLEBI can raise the statistically lowest bending strength, σ_s_ (94.3 MPa), at *P*_a_ = 0 with a high correlation coefficient. This is because, although higher than the other experimental conditions, the weakest sample of the 215 kGy data set had a much lower σ_b_ than the others. When the single weakest sample in each data set was omitted, σ_s_ was higher in the 215 kGy samples than the untreated and the 43, 129, 301, and 430 kGy samples.
3. Interestingly, electron spin resonance (ESR) of the CF showed that naturally occurring dangling bonds were reduced by 43 kGy but sharply increased at 129 kGy and 215 kGy. As far as the authors know, this is the first time this increase is reported. The *σ*_b_ appears to be controlled by an increase or decrease in dangling bond density in the CF by HLEBI.
4. Improvements are most likely from charge transfer from the activated highly conductive CF to the PC generating strong bonds, which are possibly covalent, of CF:C:O:C:PC and CF:C:C:PC at the CF/PC interface. The 215 HLEBI treatment enhanced adhesion to PC around the CF circumference, along with preventing fiber pull-out and ply delamination. The CF itself is also strengthened by the HLEBI. However, carefulness is highly recommended in each situation when applying practically, since higher doses can lower the strength of the composite.

## Data Availability

Data are available from the corresponding author upon request.
